# The relationship between human leukocyte antigen-DP/DQ gene polymorphisms and the outcomes of HCV infection in a Chinese population

**DOI:** 10.1186/s12985-017-0901-7

**Published:** 2017-12-06

**Authors:** Peng Huang, Haozhi Fan, Ting Tian, Peiwen Liao, Jun Li, Rongbin Yu, Xueshan Xia, Yue Feng, Jie Wang, Yuan Liu, Yun Zhang, Ming Yue

**Affiliations:** 1 Department of Epidemiology and Biostatistics, School of Public Health, Nanjing Medical University, Nanjing, 211166 China; 2 Institute of Epidemiology and Microbiology, Huadong Research Institute for Medicine and Biotechnics, Nanjing, China; 3 Pubilc Health Sciences Department, Karolinska Institute, Solna, Sweden; 4 Department of Infectious Diseases, the First Affiliated Hospital of Nanjing Medical University, Nanjing, 210029 China; 5 Faculty of Life Science and Technology, Kunming University of Science and Technology, Yunnan, 650500 China; 6 Department of Basic and Community Nursing, School of Nursing, Nanjing Medical University, Nanjing, 211166 China

**Keywords:** Hepatitis C virus, HLA, Gene polymorphism, Drug use, Hemodialysis, Paid blood donors

## Abstract

**Background:**

Recently, human leukocyte antigen (*HLA*) class-II gene polymorphisms have been reported to be related to Hepatitis C virus (HCV) infection and chronicity. The objective of this study was to explore the relationship of *HLA-DP* rs9277535 and *HLA-DQ* rs7453920 with the outcomes of HCV infection.

**Methods:**

The rs9277535 and rs7453920 were genotyped in 370 subjects with chronic HCV infection, 194 subjects with spontaneous HCV clearance, and 973 subjects with non-HCV infection from the Chinese population using the ABI TaqMan allelic discrimination assay.

**Results:**

Logistic regression analyses showed that the minor allele A of rs7453920 significantly increased the susceptibility of HCV infection in dominant model (adjusted OR = 1.33, 95% CI: 1.04–1.71, *P* = 0.026) and additive models (adjusted OR = 1.30, 95% CI: 1.06–1.60, *P* = 0.012). Rs9277535 A allele significantly increased the risk of chronic HCV infection in dominant model (adjusted OR = 1.52, 95% CI: 1.01–2.28, *P* = 0.046). Haplotype AA showed a higher risk of HCV infection than the most frequent haplotype GG (adjusted OR = 1.37, 95% CI: 1.05–1.78, *P* = 0.018).

**Conclusion:**

The *HLA-DQ* rs7453920 and *-DP* rs9277535 mutations were significantly associated with HCV infection susceptibility and chronicity, respectively.

**Electronic supplementary material:**

The online version of this article (10.1186/s12985-017-0901-7) contains supplementary material, which is available to authorized users.

## Background

Hepatitis C virus (HCV) infection remains a major public health concern, although there has been significant progress in the treatment of HCV infection due to the presence of direct antiviral drugs (DAAs). Based on the estimation of the World Health Organization (WHO), there are 130–150 million people infected with HCV globally, and about 30% of infected people will be spontaneously clear, while the others will develop chronic infection, and further progress to cirrhosis and hepatocellular carcinoma (HCC) [1]. In China, the average prevalence of HCV infection is about 3.2%, a total of about 29 million hepatitis C virus carriers [1]. The pathogenesis of HCV infection and chronicity are elusive, but biological characteristics of HCV, host immunity, genetic background and environmental behavior factors are deemed to be interactively involved with the complex pathogenesis [2–4].

The innate and adaptive immune response after the infection of HCV, which varies across individuals, affect the susceptibility and chronicity to HCV infection [5, 6]. The human leukocyte antigen (HLA) system, as the major histocompatibility complex (MHC) in human, plays an important role in specific immune response and immune regulation through the interaction with natural killer (NK) cells, T- and B- lymphocytes and cytokines [7, 8]. HLA molecules are categorized into three basic groups: class I, class II, and class III, which genes are located close together on the short arm of chromosome 6 [9].

It is globally recognized that HLA class II molecules, categorized into three sub-regions: *HLA-DR, −DQ* and *-DP*, are key determinants in the immune response to HCV infection through the effective presentation of T-cells to viral antigens [10, 11]. Researchers have recently discovered that variation in *HLA* class II genes are determinants of the susceptibility and chronicity of HCV infection. For example, a comparative review showed that HLA DR*13 alleles were protective against HCV infections in several populations [12]. Katayoun et al. conducted a genome wide association study (GWAS) and found the HLA-DRB1 * 0301 alleles were significantly associated with viral clearance, and DQA1 * 0201 and DQB1 * 0602 alleles are significantly associated with HCV persistence [13].

It has been studied that the single nucleotide polymorphism (SNP) rs9277535 of *HLA-DP* is associated with systemic lupus erythematosus (SLE) susceptibility, cervical cancer susceptibility, and persistent hepatitis B virus (HBV) infection, the SNP rs7453920 of *HLA-DQ* is also suggested to be associated with ankylosing spondylitis (AS) susceptibility, persistent HBV infection [14–19]. However, the evidence is sparse about whether above two SNPs is related to HCV in a Chinese population. Therefore, we investigate the relationship of the *HLA-DP* rs9277535 and *-DQ* rs7453920 with the susceptibility and chronicity of HCV infection in the Chinese population.

## Methods

### Participants

A total of 437 consecutive participants of drug users were recruited from the Nanjing compulsory drug rehabilitation center (Nanjing, Jiangsu, China) between May 2006 and Dec 2009, 714 consecutive participants of hemodialysis (HD) were recruited from nine hospital hemodialysis centers in southern China between Nov 2008 and Dec 2009, and 386 consecutive participants of paid blood donors (PBD) were recruited from Danyang (Danyang, Jiangsu, China) in Apr 2011, respectively. Cases would be excluded if they were affected or lived with any other hepatotropic virus or human immunodeficiency virus (HIV) co-infection, any other types of liver diseases (autoimmune hepatitis, metabolic disorder, alcohol liver and drugs-induced liver injury), or previous interferon and/or ribavirin therapy. All participants were grouped as follows: (1) Group A: 370 subjects with chronic HCV infection, who were anti-HCV antibodies seropositive and HCV-RNA seropositive, (2) Group B: 194 subjects with spontaneous HCV clearance, who were anti-HCV antibodies seropositive and HCV-RNA seronegative, and (3) Group C: 973 subjects with non-HCV infection, who were anti-HCV antibodies seronegative and HCV-RNA seronegative. All patients were diagnosed by experienced physicians based on clinical interviews, laboratory results and international standards. Structured interviews and standardized questionnaires were utilized to collect demographic and environmental exposure history information. Blood samples (~ 5 mL) were then collected for further virus detection, serological testing, and host DNA genotyping.

Each participates was recruited prior informed consent, agreed to participate in this study and sample collection. The study followed the Helsinki Declaration and was approved by the Institutional Review Board of Nanjing Medical University (Nanjing, China).

### Serological testing

The serological status of anti-HCV antibodies of the subjects was detected by the third-generation enzyme-linked immunosorbent assay (ELISA) (Architect Anti-HCV assay, Abbott Laboratories, Abbott Park, IL, USA). HCV RNA was extracted from plasma using kit (Cobas TaqMan HCV Test, Roche Diagnostics, Manheim, Germany) and HCV genotyping was performed by the Murex HCV serotype ELISA kit (Abbott, Wiesbaden, Germany). All tests were conducted in accordance with the manufacturer’s instructions.

### Genotyping assays

Genomic DNA was extracted from peripheral blood samples by protease K digestion, phenol-chloroform extraction and ethanol precipitation, and the DNA concentration was tested on NanoDrop 2000 spectrophotometer (Thermo Scientific, DE). According to the relevant HLA class II single nucleotide polymorphism (SNP) literature, two SNPs were selected as candidate sites, including *HLA-DP*: rs9277535 and *HLA-DQ*: rs7453920, which showed minor allele frequency (MAF) > 5% in Chinese Han population in NCBI dbSNP database (http://www.ncbi.nlm.nih.gov/SNP). SNP rs9277535 and rs7453920 were genotyped using the TaqMan allele identification assay on the ABI PRISM 7900HT system (Applied Biosystems, Foster City, CA, USA). Rs9277535 and rs7453920 specific TaqMan probes, forward and reverse primer information is shown in Additional file 1: Table S1. All genotyping was performed blindly in the context that the operator did not know the clinical data of the subject. All genotyping assays were conducted with blinded to the subject’s clinical data. In each of the 384-well plates, two blank controls (water) were used for quality control, and randomly selected 10% of the samples were submitted to repeated experiments, each SNP yield 100% consistency. The success rates of the two SNP genotypes were above 94%.

### Statistical analysis

The distributions of demographic and clinical data between groups were obtained by the one-way ANOVA (for variance homogeneity) or Welch (for variance) and chi-square (χ2) test. Hardy-Weinberg equilibrium (HWE) was assessed in three groups by a goodness-of-fit χ2 tests. The odds ratio (OR) and 95% confidence interval (CI) were calculated to estimate the natural susceptibility and long-term risk of SNP and HCV infection by binary logistic regression analysis, with adjusting for age, sex, high-risk population, alanine aminotransferase (ALT), aspartate aminotransferase (AST), and HCV genotypes. The corresponding OR and their 95% CI were calculated using the co-dominant model, the dominant model and the additive model, respectively. The parameters D’ and R^2^ were calculated using the Haploview software (version 4.2) to analyze whether the two studied SNP linkage disequilibrium (LD). Single haplotype analysis was performed utilizing PHASE software (version 2.1) based on the observed genotype. All statistical analyses were performed using stata13.0. *P* < 0.05 in a two-sided test was considered statistically significant.

## Results

### Demographic and clinical characteristics

The demographic and clinical characteristics of the 370 subjects with chronic HCV infection, 194 subjects with spontaneous HCV clearance, and 973 subjects with non-HCV infection were set out in Table 1. Distributions of age and gender were comparable among the three groups (*P* = 0.090 and 0.860, respectively). However, there were significantly different distributions of ALT, AST, high-risk population, and HCV genotypes in the three groups (all *P* < 0.001).

**Table 1:** Demographic and clinical characteristics in subjects

Variables	Group A (%)	Group B (%)	Group C (%)	*P*
	*n* = 370	*n* = 194	*n* = 973	
Mean age, year (SD)	46.86 ± 13.05	47.03 ± 11.26	48.43 ± 13.73	0.090^a^
Age ≥ 50 (%)	157 (22.6)	74 (10.7)	463 (66.7)	0.026^b^
Gender				0.860^b^
Male	204 (55.1)	110 (56.7)	531 (54.6)	
Female	166 (44.9)	84 (43.3)	442 (45.4)	
ALT ≥40 U/L	104 (28.1)	27 (13.9)	48 (4.9)	<0.001^c^
AST ≥ 40 U/L	128 (34.6)	30 (15.5)	80 (8.2)	<0.001^c^
High-risk population				<0.001^b^
Drug user	155 (41.9)	36 (18.6)	246 (25.3)	
HD	74 (20.0)	89 (45.9)	551 (56.6)	
PBD	141 (38.1)	69 (35.6)	176 (18.1)	
HCV genotypes				<0.001^b^
1	236 (63.8)	114 (58.8)	–	
Non-1	51 (13.8)	53 (27.3)	–	
Mixed	83 (22.4)	27 (13.9)	–	

Group A: chronic HCV infection; Group B: spontaneous HCV clearance; Group C: non-HCV infection; Non-1: genotype 2, 3 and unknown; Mixed: co-infected with genotype 1/ 2/3

*Abbreviations*: ALT, alanine transaminase; AST, aspartate transaminase; SD, standard deviation; HD, hemodialysis patient; PBD, paid blood donors

^a^ *P* value of Welch among three groups, heterogeneity of variance

^b^ *P* value of χ^2^-test among three/two groups

^c^ *P* value of Kruskal-Wallis test or Mann-Whitney U test among three/two groups

Observed genotype frequencies for rs9277535 and rs7453920 in non-HCV infection group were all in Hardy-Weinberg equilibrium (*P* = 0.107 for rs9277535, *P* = 0.114 for rs9277535, respectively).

### Association of candidate SNPs with HCV susceptibility and chronicity

Compared with the spontaneous HCV clearance group (group B), logistic regression analysis showed that the minor allele A of HLA-DP rs9277535 significantly increased the risk of chronic HCV infection in dominant model (adjusted OR = 1.52, 95% CI: 1.01–2.28, *P* = 0.046), after adjusting for gender, age, ALT, AST, high-risk population, and HCV genotypes (Table 2).

**Table 2:** Association of *HLA-DP/DQ* with HCV susceptibility and clearance

SNPs (genotype)	Group A	Group B	Group C	Group(A + B)/Group C		Group A/Group B	
	n (%)	n (%)	n (%)	OR^a^	*P* ^a^	OR^b^	*P* ^b^
*HLA-DP* rs9277535							
GG	96 (26.1)	64 (33.2)	276 (28.7)	1.00	–	1.00	–
AG	190 (51.6)	83 (43.0)	455 (47.3)	1.02 (0.79–1.33)	0.876	**1.60 (1.03–2.48)**	**0.035**
AA	82 (22.3)	46 (23.8)	231 (24.0)	1.00 (0.73–1.37)	0.989	1.35 (0.81–2.27)	0.250
Dominant model				1.01 (0.79–1.30)	0.906	**1.52 (1.01–2.28)**	**0.046**
Additive model				1.00 (0.86–1.17)	0.979	1.19 (0.91–1.54)	0.198
*HLA-DQ* rs7453920							
GG	237 (69.3)	119 (66.1)	671 (72.4)	1.00	–	1.00	–
AG	84 (24.6)	55 (30.6)	228 (24.6)	1.26 (0.97–1.65)	0.084	0.90 (0.58–1.39)	0.643
AA	21 (6.1)	6 (3.3)	28 (3.0)	**1.84 (1.03–3.29)**	**0.040**	1.96 (0.71–5.37)	0.193
Dominant model				**1.33 (1.04–1.71)**	**0.026**	1.00 (0.66–1.52)	0.989
Additive model				**1.30 (1.06–1.60)**	**0.012**	1.10 (0.78–1.54)	0.599

Group A: chronic HCV infection; Group B: spontaneous HCV clearance; Group C: non-HCV infection; Group (A + B): HCV-infected patients

*Abbreviations*: *SNPs* single nucleotide polymorphisms

Bold type indicates statistically significant results

^a^The *P* value, odds ratio (OR), 95% confidence intervals (CI) of Group (A + B) versus Group C were calculated on the basis of the logistic regression model, adjusted by gender, age, ALT, AST, and high-risk population

^b^The *P* value, odds ratio (OR), 95% confidence intervals (CI) of Group A versus Group B were calculated on the basis of the logistic regression model, adjusted by gender, age, ALT, AST, high-risk population, and HCV genotypes

Compared with the non-HCV infection group, logistic regression analysis showed that the minor allele A of HLA-DQ rs7453920 significantly increased the susceptibility of HCV infection in dominant model (adjusted OR = 1.33, 95% CI: 1.04–1.71, P = 0.026) and additive model (adjusted OR = 1.30, 95% CI: 1.06–1.60, *P* = 0.012), adjusted by gender, age, ALT, AST, and high-risk population. But there was no evidence that there was a significant association between HLA-DP rs9277535 variant genotype and HCV susceptibility (Table 2).

### Stratified analysis

In order to control the bias due to gender, age, high-risk population, and viral genotypes in each population, we further performed the stratified analysis. The results of the stratified analysis are presented in Table 3. Dominant models were employed in stratified analysis for each SNP. Logistic regression analysis showed that rs9277535 variant genotypes significantly increased risk of chronic HCV infection among people less than 50 years old (adjusted OR = 1.71, 95% CI: 1.02–2.86, P = 0.042) and among PBD population (adjusted OR = 1.94, 95% CI: 1.04–3.63, *P* = 0.037). While rs7453920 variant genotypes significantly increased the susceptibility of HCV infection among people less than 50 years old (adjusted OR = 1.42, 95% CI: 1.02–1.98, *P* = 0.040) and HD population (adjusted OR = 1.53, 95% CI: 1.04–2.25, *P* = 0.029). However, no significant association of rs9277535 and rs7453920 variant genotypes with HCV susceptibility and chronicity was observed in other strata (all P > 0.05) (Additional file 1: Table S2 and S3).

**Table 3:** Stratified analysis the association of *HLA-DP/DQ* with HCV susceptibility and chronicity

SNP	Subgroups	Group(A + B)/Group C		Group A/Group B	
		OR^a^	*P* ^a^	OR^b^	*P* ^b^
rs9277535	Age				
	<50	1.02(0.74–1.42)	0.901	**1.71(1.02–2.86)**	**0.042**
	≥50	1.01(0.69–1.47)	0.965	1.29(0.64–2.57)	0.475
	High-risk population				
	Drug user	0.92(0.58–1.47)	0.733	2.19(0.94–5.11)	0.069
	HD	1.35(0.89–2.04)	0.162	0.87(0.42–1.81)	0.700
	PBD	0.93(0.60–1.45)	0.746	**1.94(1.04–3.63)**	**0.037**
rs7453920	Age				
	<50	**1.42(1.02–1.98)**	**0.040**	0.82(0.49–1.37)	0.445
	≥50	1.21(0.82–1.78)	0.329	1.23(0.58–2.58)	0.591
	High-risk population				
	Drug user	1.36(0.83–2.22)	0.218	1.22(0.45–3.32)	0.693
	HD	**1.53(1.04–2.25)**	**0.029**	1.02(0.51–2.01)	0.962
	PBD	1.20(0.75–1.93)	0.449	0.83(0.43–1.60)	0.572

Group A: chronic HCV infection; Group B: spontaneous HCV clearance; Group C: non-HCV infection; Group (A + B): HCV-infected patients

Abbreviations: HD, hemodialysis patient; PBD, paid blood donors

Bold type indicates statistically significant results

^a^The *P* value, OR and 95% CIs of group (A + B) versus Group C were calculated on the basis of the binary logistic regression model, adjusted by gender, age, ALT, AST, and high-risk population in dominant model (GG versus AG + AA for rs9277535 and rs7453920)

^b^The *P* value, OR and 95% CIs of group A versus Group B were calculated on the basis of the binary logistic regression model, adjusted by gender, age, ALT, AST, high-risk population, and HCV genotypes in dominant model (GG versus AG + AA for rs9277535 and rs7453920)

### Haplotype analysis

The D’ value and R^2^ value were used for quantifying the level of LD, and found no significant LD between rs9277535 and rs7453920 (D’ = 0.131, R^2^ = 0.004). To further evaluate the effects of HLA-DP and HLA-DQ polymorphism on HCV infection outcomes, which consisted of rs9277535 and rs7453920 variant alleles, we performed haplotype analysis (Table 4). When compared with the most frequent GG haplotype, the haplotype carrying rs3077-A and rs2856718-A (AA) showed increased the susceptibility of HCV infection (adjusted OR = 1.37, 95% CI: 1.05–1.78, *P* = 0.018).

**Table 4:** Haplotypes analysis of rs9277535 and rs7453920 with different HCV outcomes

Haplotype^a^	Group A(%)	Group B(%)	Group C(%)	Group (A + B)/Group C		Group A/Group B	
	*n* = 740	*n* = 388	*n* = 1946	OR^b^	*P* ^b^	OR^c^	*P* ^c^
GG	345 (46.6)	191 (49.2)	929 (47.7)	1.00	1.00	1.00	1.00
GA	39 (5.3)	21 (5.4)	92 (4.7)	1.07 (0.74–1.54)	0.712	0.86 (0.48–1.56)	0.628
AG	269 (36.4)	130 (33.5)	733 (36.7)	0.93 (0.78–1.10)	0.390	1.18 (0.88–1.58)	0.278
AA	87 (11.8)	46 (11.9)	192 (9.9)	**1.37 (1.05–1.78)**	**0.018**	1.09 (0.71–1.66)	0.698

Group A: chronic HCV infection; Group B: spontaneous HCV clearance; Group C: non-HCV infection; Group (A + B): HCV-infected patients

*Abbreviations*: *SNPs* single nucleotide polymorphisms

Bold type indicates statistically significant results

^a^Order of single nucleotide polymorphisms comprising the HLA class II haplotypes: s9277535 and rs7453920

^b^The *P* value, OR and 95% CIs of group (A + B) versus Group C were calculated on the basis of the binary logistic regression model, adjusted by gender, age, ALT, AST, and high-risk population

^c^The *P* value, OR and 95% CIs of group A versus Group B were calculated on the basis of the binary logistic regression model, adjusted by gender, age, ALT, AST, high-risk population, and HCV genotypes

## Discussion

It is well known that HCV is a serious public health problem. Studies on the relationship between HLA Class II gene polymorphisms and the susceptibility and chronicity of HCV infection help us better understand the complicated mechanism. The current research found that *HLA-DP* rs9277535 A and *-DQ* rs7453920 A allele significantly increased the risk of HCV chronicity and HCV infection in Chinese Han population, respectively.

The HLA determinant as the principal component of immunogenicity factors has been found associated with HCV infection [20, 21]. HLA-DQ belongs to HLA Class II molecules play crucial roles in the regulation of immune responses [22]. HLA-DQ molecules, which are induced when *HLA-DQ* genes are expressed, play a regulatory role by binding to exogenous antigens and presenting exogenous substances to CD4+ T lymphocytes [23, 24]. A previous study detected genetic variants of rs2856718 in intron region of *HLA-DQ* genes also strongly associated with the susceptibility of HCV infection in Chinese population [25]. The current study found that the individuals carrying *HLA-DQ* rs7453920 A allele had a statistically significantly higher risk of HCV infection. It is possible that rs7453920 variation may alter the expression of the *HLA-DQ* gene with alternation of non-coding RNA sequences, further affecting the recognition and presentation of exogenous antigen peptide, thus leading to effects on the secretion of cytokines and the differentiation of T-cells. The study of Xu et al. showed that the rs7453920G allele was a risk factor for HBV infection, while rs7453920A allele served as a protective factor in chronic Hepatitis C [26]. Furthermore, rs7453920A allele could predict the better prognosis in liver transplant recipients, and was associated with decreased risk of ankylosing spondylitis (AS) [27]. The explanation of this could be that HBV and HCV have remarkable differences in the molecular virology and specific immune responses, although both of them are hepatotrophic factors and tend to produce liver diseases. For instance, HLA DQB1*0301and HLA DRB1*11 play a protective role in HCV infections, but the opposite in persistent HBV infection [12].

Previous research has shown that low viral diversity and strong CD4+ T-cell response targeting a broad range of virus antigenic epitopes are connection with spontaneous viral clearance (SVC), while infectious agents can lead to incompetent T-cell response and persistent infection through a variety of complex mechanisms [28]. Furthermore, the epitopes of CD4+ T-cell are highly promiscuous and can often be restricted by a variety of different HLA class II molecules [29]. Genetic association studies showed that HLA class II-restricted epitopes goes unrecognized by T-cells may be one of the possible mechanisms of HCV to avoid immune clearance [30]. A meta-analysis confirmed that DQB1*02, DQB1*03, DRB1*04 and DRB1*11 were associated with spontaneous hepatitis C virus clearance [31]. The current study revealed that the individuals carrying *HLA-DP* rs9277535 A allele had a statistically significantly higher risk of chronic HCV infection. Previous research has shown that *HLA-DP* rs9277535A allele might protect against chronic HBV infection in Japanese, Korean, Taiwanese, and Thai populations, but might increase the risk of chronic HBV infection in Han Chinese and Saudi Arabian populations [32]. Other studies have found rs9277535A allele that could decrease SLE susceptibility, however, was associated with increased cervical cancer susceptibility in Chinese females [17, 18]. Rs9277535 may be the binding site of some microRNA (miRNA), where 3′-UTR region of *HLA- DPB1* gene is found. Research indicates that miRNA affects the stability and translation of messenger RNA (mRNA) by base-pairing with complementary sequences within mRNA molecules [33]. Thus, rs9277535 may affect the regulatory function of miRNA by disrupting microRNA-mRNA interaction, resulting in the deregulation of the expression and stability of HLA mRNA.

The study indicated that the association between *HLA-DP/DQ* and the susceptibility and chronicity of HCV infection was likely to be more pronounced among people less than 50 years old, PBD population and HD population. It was indicated in the previous research that chronic HD patients were with renal failure and compromised immune systems, and were less likely to clear HCV and other infection [34–36]. Therefore, we can deduce that HD patients carrying *HLA-DP* rs7453920 A allele were more prone to gain the genetic benefit because of severe alterations in immune systems. The current research also suggested that the haplotype AA showed a higher risk of HCV infection than the most frequent haplotype GG, which was consistent with the rs7453920 effect but failed to show in rs9277535 effect. This indicates that the effects of *HLA-DP* and *HLA-DQ* may not be independent, which has been proposed in a recent study [37]. Therefore, further fine mapping studies are recommended.

The studies generate the genetic evidence of association between *HLA Class II* gene polymorphisms and the outcome of HCV infection, which may facilitate scientists and clinicians to identify new targets for therapy and personalized medical-based strategy along with other SNP information. There are some limitations in this study. First, the participants of this study were drug addicts, HD and PBD, which have impaired immune function and thus may not represent all subjects with HCV persistent infection and spontaneous clearance in the general population. Second, the clinical information including the infection time of HCV and immunization status for each individual was not sufficient, demonstrating potential biases arising from unmeasured confounding variables. Finally, the sample size of HCV persistent infection group (*n* = 370) and spontaneous clearance group (*n* = 194) were relatively small, further research on a large scale is therefore necessary.

## Conclusions

In conclusion, this study found that HLA-DQ rs7453920 A allele may be a genetic factor of HCV infection, while HLA-DP rs9277535 A allele may be for chronic HCV infection in the Chinese Han population.

## Additional file

Additional file 1:
**Table S1.** Information of primers and probes for TaqMan allelic discrimination. **Table S2.** Stratified analysis the association of *HLA-DP* rs9277535 with HCV clearance. **Table S3.** Stratified analysis the association of *HLA-DQ* rs7453920 with HCV susceptibility. (DOCX 32 kb)

## Abbreviations

ALT: Alanine aminotransferase
AS: Ankylosing spondylitis
AST: Aspartate aminotransferase
CI: Confidence interval
DAAs: Direct antiviral drugs
DCs: Dendritic cells
ELISA: Third-generation enzyme-linked immunosorbent assay
HCC: Hepatocellular carcinoma
HCV: Hepatitis C virus
HD: Hemodialysis
HIV: Human immunodeficiency virus
HLA: Human leukocyte antigen
HWE: Hardy-Weinberg equilibrium
LD: Linkage disequilibrium
MAF: Minor allele frequency
MHC: Major histocompatibility complex
miRNA: microRNA
mRNA: Messenger RNA
OR: Odds ratio
PBD: Paid blood donors
SLE: Systemic lupus erythematosus
SNP: Single nucleotide polymorphism
SVC: Spontaneous viral clearance
WHO: World Health Organization
