# Supplementary material for: The relationship between human leukocyte antigen-DP/DQ gene polymorphisms and the outcomes of HCV infection in a Chinese population
Source: Virol J. 2017 Dec 6;14:235. doi: 10.1186/s12985-017-0901-7 (PMC5719872; doi:10.1186/s12985-017-0901-7)
Supplement: Additional file 1: — Table S1. Information of primers and probes for TaqMan allelic discrimination. Table S2. Stratified analysis the association of HLA-DP rs9277535 with HCV clearance. Table S3. Stratified analysis the association of HLA-DQ rs7453920 with HCV susceptibility. (DOCX 32 kb) [file 12985_2017_901_MOESM1_ESM.docx]

Additional file 1: Table S1. Information of primers and probes for TaqMan allelic discrimination

| SNPs | Allele | Nearby gene | MAF^a/b^ | TaqMan-MGB probe/primers sequences (5’-3’) |
| --- | --- | --- | --- | --- |
| rs9277535 | G>A | *HLA-DP* | 0.477/0.463 | Forward primer : AATGGTGAGCAGACTGCAAATCT |
|  |  |  |  | Reverse primer : TGGTAATGATAAAACATGCTCTCAGTAA |
|  |  |  |  | Probe-T: FAM- ATAGGACCCGTATTC-MGB |
|  |  |  |  | Probe-C: HEX- ATAGGACCCATATTC-MGB |
| rs7453920 | G>A | *HLA-DQ* | 0.153/0.195 | Forward primer : TTTAGGGAGGTAAGAGGGAAAGC |
|  |  |  |  | Reverse primer : CGAGAACGCCCTGATCTAAGA |
|  |  |  |  | Probe-T: FAM-ACCGATTCGACATTG-MGB |
|  |  |  |  | Probe-C: HEX-CCGATTCAACATTG-MGB |

Abbreviations: SNPs, single nucleotide polymorphisms; MAF, minor allele frequency.

a minor allele frequencies in control group.

b minor allele frequencies from HapMap of Han Chinese in Beijing, China (CHB) (dbSNP, build128; available at <http://www.ncbi.nlm.nih.gov/SNP/>).

**Table S2. Stratified analysis the association of *HLA-DP* rs9277535 with HCV clearance**

| Subgroups | Group A n (%) | | | Group B n (%) | | | Group C n (%) | | | OR(95%CI)^a^ | *P^a’^* | | OR(95%CI)^b^ | *P^b^* | |
| --- | --- | --- | --- | --- | --- | --- | --- | --- | --- | --- | --- | --- | --- | --- | --- |
|  | GG | AG | AA | GG | AG | AA | GG | AG | AA |  | | |  | | |
| Age |  |  |  |  |  |  |  |  |  |  | |  |  | |  |
| ＜50 | 50(23.6) | 115(54.3) | 47(22.2) | 42(35.0) | 50(41.7) | 28(23.3) | 148(29.5) | 248(49.4) | 106(21.1) | 1.02(0.74-1.42) | | 0.901 | **1.71(1.02-2.86)** | | **0.042** |
| ≥50 | 46(29.5) | 75(48.1) | 35(22.4) | 22(30.1) | 33(45.2) | 18(24.7) | 128(27.8) | 207(45.0) | 125(27.2) | 1.01(0.69-1.47) | | 0.965 | 1.29(0.64-2.57) | | 0.475 |
| Gender |  |  |  |  |  |  |  |  |  |  | |  |  | |  |
| Male | 56(27.7) | 101(50.0) | 45(22.3) | 38(34.9) | 46(42.2) | 25(22.9) | 162(31.0) | 238(45.5) | 123(23.5) | 1.03(0.74-1.42) | | 0.870 | 1.52(0.90-2.58) | | 0.117 |
| Female | 40(24.1) | 89(53.6) | 37(22.3) | 26(31.0) | 37(44.0) | 21(25.0) | 114(26.0) | 217(49.4) | 108(24.6) | 1.00(0.69-1.46) | | 0.988 | 1.39(0.71-2.71) | | 0.338 |
| High-risk population | |  |  |  |  |  |  |  |  |  | |  |  | |  |
| Drug user | 40(26.0) | 86(55.8) | 28(18.2) | 13(36.1) | 12(33.3) | 11(30.6) | 61(24.8) | 128(52.0) | 57(23.2) | 0.92(0.58-1.47) | | 0.733 | 2.19(0.94-5.11) | | 0.069 |
| HD | 20(27.4) | 31(42.5) | 22(30.1) | 22(25.0) | 44(50.0) | 22(25.0) | 161(29.8) | 243(45.0) | 136(25.2) | 1.35(0.89-2.04) | | 0.162 | 0.87(0.42-1.81) | | 0.700 |
| PBD | 36(25.5) | 73(51.8) | 32(22.7) | 29(42.0) | 27(39.1) | 13(18.9) | 54(30.7) | 84(47.7) | 38(21.6) | 0.93(0.60-1.45) | | 0.746 | **1.94(1.04-3.63)** | | **0.037** |
| HCV genotypes | |  |  |  |  |  |  |  |  |  | |  |  | |  |
| 1 | 52(24.4) | 116(54.5) | 45(21.1) | 42(31.1) | 59(43.7) | 34(25.2) | -- | -- | -- | -- | | -- | 1.39(0.83-2.33) | | 0.216 |
| Non-1 | 18(23.4) | 38(49.4) | 21(27.2) | 10(38.5) | 11(42.3) | 5(19.2) | -- | -- | -- | -- | | -- | 2.16(0.77-6.02) | | 0.143 |
| Mixed | 26(33.3) | 36(46.2) | 16(20.5) | 12 (37.5) | 13(40.6) | 7(21.9) | -- | -- | -- | -- | | -- | 1.27(0.45-3.60) | | 0.653 |

Group A: chronic HCV infection; Group B: spontaneous HCV clearance; Group C: non-HCV infection; Group (A+B): HCV-infected patients.

Abbreviations: HD, hemodialysis patient; PBD, paid blood donors.

Bold type indicates statistically significant results.

^a^ The *P* value, OR and 95% CIs of group (A+B) versus Group C were calculated on the basis of the binary logistic regression model, adjusted by gender, age, ALT, AST, and high-risk population in dominant model (GG versus AG+AA for rs9277535).

^b^ The *P* value, OR and 95% CIs of group A versus Group B were calculated on the basis of the binary logistic regression model, adjusted by gender, age, ALT, AST, high-risk population, and HCV genotypes in dominant model(GG versus AG+AA for rs9277535).

**Table S3. Stratified analysis the association of *HLA-DQ* rs7453920 with HCV susceptibility**

| Subgroups | Group A n (%) | | | Group B n (%) | | | Group C n (%) | | | OR(95%CI)^a^ | *P^a’^* | | OR(95%CI)^b^ | *P^b^* |
| --- | --- | --- | --- | --- | --- | --- | --- | --- | --- | --- | --- | --- | --- | --- |
| (Genotypes) | GG | GA | AA | GG | GA | AA | GG | GA | AA |  | | |  | |
| Age |  |  |  |  |  |  |  |  |  |  | |  |  |  |
| ＜50 | 141(69.5) | 54(26.6) | 8(3.9) | 72(63.7) | 36(31.9) | 5(4.4) | 359(74.2) | 115(23.8) | 10(2.1) | **1.42(1.02-1.98)** | | **0.040** | 0.82(0.49-1.37) | 0.445 |
| ≥50 | 96(69.1) | 30(21.6) | 13(9.3) | 47(70.1) | 19(28.4) | 1(1.5) | 312(70.4) | 113(25.5) | 18(4.1) | 1.21(0.82-1.78) | | 0.329 | 1.23(0.58-2.58) | 0.591 |
| Gender |  |  |  |  |  |  |  |  |  |  | |  |  |  |
| Male | 132(68.4) | 54(28.0) | 7(3.6) | 66(63.5) | 34(32.7) | 4(3.8) | 364(70.7) | 140(27.2) | 11(2.1) | 1.29(0.93-1.79) | | 0.127 | 0.83(0.49-1.41) | 0.494 |
| Female | 105(70.5) | 30(20.1) | 14(9.4) | 53(69.7) | 21(27.6) | 2(2.6) | 307(74.5) | 88(21.4) | 17(4.1) | 1.38(0.94-2.04) | | 0.102 | 1.39(0.68-2.86) | 0.367 |
| Routes of infection | |  |  |  |  |  |  |  |  |  | |  |  |  |
| Drug use | 90(68.7) | 28(21.4) | 13(9.9) | 21(75.0) | 6(21.4) | 1(3.6) | 159(74.3) | 43(20.1) | 12 (5.6) | 1.36(0.83-2.22) | | 0.218 | 1.22(0.45-3.32) | 0.693 |
| Hemodialysis | 46(63.9) | 22(30.6) | 4(5.5) | 53(61.6) | 28(32.6) | 5(5.8) | 384(70.7) | 146(26.9) | 13(2.4) | **1.53(1.04-2.25)** | | **0.029** | 1.02(0.51-2.01) | 0.962 |
| PDD | 101(72.7) | 34(24.4) | 4(2.9) | 45(68.2) | 21(31.8) | 0(0) | 128(75.3) | 39(22.9) | 3(1.8) | 1.20(0.75-1.93) | | 0.449 | 0.83(0.43-1.60) | 0.572 |
| HCV genotypes | |  |  |  |  |  |  |  |  |  | |  |  |  |
| 1 | 138(70.8) | 45(23.1) | 12(6.1) | 82(64.6) | 40(31.5) | 5(3.9) | -- | -- | -- | -- | | -- | 0.83(0.49-1.40) | 0.487 |
| Non-1 | 45(62.5) | 22(30.6) | 5(6.9) | 15(62.5) | 9(37.5) | 0(0) | -- | -- | -- | -- | | -- | 1.32(0.46-3.79) | 0.602 |
| Mixed | 54(72.0) | 17(22.7) | 4(5.3) | 22(75.9) | 6(20.7) | 1(3.4) | -- | -- | -- | -- | | -- | 0.69(0.16-3.06) | 0.629 |

Group A: chronic HCV infection; Group B: spontaneous HCV clearance; Group C: non-HCV infection; Group (A+B): HCV-infected patients.

Abbreviations: HD, hemodialysis patient; PBD, paid blood donors.

Bold type indicates statistically significant results.

a The P value, OR and 95% CIs of group (A+B) versus Group C were calculated on the basis of the binary logistic regression model, adjusted by gender, age, ALT, AST, and high-risk population in dominant model (GG versus AG+AA for rs7453920).

b The P value, OR and 95% CIs of group A versus Group B were calculated on the basis of the binary logistic regression model, adjusted by gender, age, ALT, AST, high-risk population, and HCV genotypes in dominant model (GG versus AG+AA forrs7453920).
